# Supplementation of H7N9 Virus-Like Particle Vaccine With Recombinant Epitope Antigen Confers Full Protection Against Antigenically Divergent H7N9 Virus in Chickens

**DOI:** 10.3389/fimmu.2022.785975

**Published:** 2022-02-21

**Authors:** Dexin Kong, Taoran Chen, Xiaolong Hu, Shaorong Lin, Yinze Gao, Chunmei Ju, Ming Liao, Huiying Fan

**Affiliations:** ^1^ College of Veterinary Medicine, South China Agricultural University, Guangzhou, China; ^2^ Key Laboratory of Zoonosis Prevention and Control of Guangdong Province, College of Veterinary Medicine, South China Agricultural University, Guangzhou, China; ^3^ Key Laboratory of Animal Vaccine Development, Ministry of Agriculture, Guangzhou, China; ^4^ National and Regional Joint Engineering Laboratory for Medicament of Zoonosis Prevention and Control, College of Veterinary Medicine, South China Agricultural University, Guangzhou, China

**Keywords:** H7N9, virus-like particles, influenza conserved epitopes, cross-protection, T-cell immunity

## Abstract

The continuous evolution of the H7N9 avian influenza virus suggests a potential outbreak of an H7N9 pandemic. Therefore, to prevent a potential epidemic of the H7N9 influenza virus, it is necessary to develop an effective crossprotective influenza vaccine. In this study, we developed H7N9 virus-like particles (VLPs) containing HA, NA, and M1 proteins derived from H7N9/16876 virus and a helper antigen HMN based on influenza conserved epitopes using a baculovirus expression vector system (BEVS). The results showed that the influenza VLP vaccine induced a strong HI antibody response and provided effective protection comparable with the effects of commercial inactivated H7N9 vaccines against homologous H7N9 virus challenge in chickens. Meanwhile, the H7N9 VLP vaccine induced robust crossreactive HI and neutralizing antibody titers against antigenically divergent H7N9 viruses isolated in wave 5 and conferred on chickens complete clinical protection against heterologous H7N9 virus challenge, significantly inhibiting virus shedding in chickens. Importantly, supplemented vaccination with HMN antigen can enhance Th1 immune responses; virus shedding was completely abolished in the vaccinated chickens. Our study also demonstrated that viral receptor-binding avidity should be taken into consideration in evaluating an H7N9 candidate vaccine. These studies suggested that supplementing influenza VLP vaccine with recombinant epitope antigen will be a promising strategy for the development of broad-spectrum influenza vaccines.

## Introduction

In March 2013, a novel H7N9 subtype of avian influenza virus infection was discovered in human cases in China (1). Since then, the virus has spread rapidly throughout the country, leading to several waves of outbreaks. In particular, after the emergence of the highly pathogenic H7N9 avian influenza virus during the fifth wave, the H7N9 virus caused a sharp rise in human infection, resulting in 1,568 laboratory-confirmed cases and 616 deaths as of July 7, 2021 (http://www.fao.org/ag/againfo/programmes/en/empres/H7N9/situation_update.html). More importantly, some novel biological features of the H7N9 virus, such as immune escape mutations and antigenic drift were discovered in H7N9 variants (2–4). Thus, there is still a possibility of an H7N9 pandemic outbreak. The continuous evolution of the H7N9 virus poses a dual threat to public health and the poultry industry, and thus it is imperative to protect against H7N9 influenza infection.

Vaccination has been considered the most effective way to prevent and control influenza virus infection (5, 6). Available are the conventional influenza vaccine containing live attenuated vaccine, whole-virus inactivated vaccine, recombinant vector vaccine, and recombinant subunit vaccine. The current large-scale production of influenza vaccine depends on the supply of embryonated chicken eggs; it is very fragile for the timely supply of a sufficient influenza vaccine during pandemic outbreaks (7, 8). Therefore, it is necessary to develop a preferable method for the production of influenza vaccines. Virus-like particle (VLP) vaccine is one of the influenza subunit vaccines; the VLPs mimic the structural and immunological properties of a native virus but are innocuous. Thus, the preparation approach of an influenza vaccine based on VLPs is preferable (9, 10). The insect cell-baculovirus expression vector system (IC-BEVS) is widely used for the development of influenza VLP subunit vaccines owing to its unique advantages, including excellent safety, short production times, and straightforward scale-up (11–13). The production of VLPs based on insect cell suspension cultured in a bioreactor system is low cost and high yield (12, 14). Currently, several different influenza VLP constructs contain HA or a combination of HA and neuraminidase (HA-NA) and matrix protein M1. HA is the main target antigen for the development of avian influenza vaccines. Neuraminidase (NA) in influenza VLP contributes to protecting against a high-dose avian influenza virus challenge infection (15).

Current influenza vaccine immunization only induces specific immune responses against strain-matched influenza viruses. This cannot provide effective protection when the circulating viruses generate antigenic drift or a new pandemic virus emerges (4, 16, 17). Therefore, developing an appropriate vaccination strategy is a high priority to improve the crossprotection of influenza vaccines and prevent future pandemic outbreaks. One appropriate approach to improving the crossprotection of the influenza vaccine is by combining it with the toll-like receptor ligand or the influenza conserved epitopes fusion protein adjuvant. Studies have shown that immunization with influenza vaccines based on influenza conserved epitopes induces crossreaction immune responses to confer crossprotection against homologous and heterologous influenza virus challenge (18–23). Nevertheless, influenza vaccines based on influenza conserved epitopes have shown limited protection against homologous and heterologous influenza challenges in reducing signs of clinical symptoms and virus shedding (24–26). These findings indicate that fusion protein of the recombined influenza conserved epitopes can act as an adjuvant to enhance the crossprotection of influenza VLP vaccine against drifted influenza virus. Poly(I:C), a toll-like receptor (TLR)-3 ligand, is a potent adjuvant, intranasal delivery of influenza vaccine with Poly(I:C) elicited robust antigen-specific cell-mediated immune responses (27, 28). Poly(I:C) has been identified to induce strong Th1 immune responses. The induction of protective T-cell responses can enhance the crossprotection of the influenza vaccine.

The main goal of this study was to develop an influenza VLP subunit vaccine and an effective supplement vaccination strategy to provide crossprotection against an influenza virus challenge. A recombinant protein (HMN) consisting of three tandem conserved epitopes: two repeats of HA_76-130_; four repeats of M2e; and eight repeats of NP_55-69_ was constructed, and a Poly(I:C) was used as a vaccine supplement in the present study. The result demonstrated that the influenza VLP subunit vaccine induced robust HI and neutralizing antibody titers to crossprotect against challenge with a lethal homologous and heterologous H7N9 virus. The influenza VLP vaccine supplement with HMN or Poly(I:C) enhanced a Th1-biased influenza-specific immune response in chickens, which was significantly inhibited virus shedding.

## Materials and Methods

### Ethics Statement

All experiments involved in the live H7N9 avian influenza viruses (AIVs) were performed in a biosafety level 3 laboratory facility at South China Agricultural University (SCAU) (CNAS BL0011) in accordance with protocols. All animals involved in the experiments were reviewed and approved by the Institution Animal Care and Use Committee at SCAU and treated in accordance with the guidelines (2017A002).

### Cells and Viruses

*Spodoptera frugiperda* 9 (Sf9) and BTI-TN-5B1-4 (High Five™) insect cells were used in this study. Sf9 cells (Invitrogen, Waltham, MA, USA) were maintained in Sf-900 II serum-free medium (Gibco, Carlsbad, CA, USA) and used for the production of recombinant baculovirus. High Five™ cells (Invitrogen, USA) were maintained as a suspension in HF-SFM (World-Medium, Suzhou, China) in shaker flasks at a speed of 100–120 rpm and used for the production of recombinant proteins. Both insect cell lines were cultured at 27°C. Madin-Darby canine kidney (MDCK) cells were maintained at 37°C in 5% CO_2_ in Dulbecco’s modified Eagle’s medium (DMEM) supplemented with 10% (v/v) heat-inactivated fetal bovine serum (Invitrogen, USA).

HPAI H7N9 viruses A/Chicken/Guangdong/16876/2016 (H7N9-16876) (29), A/Chicken/Qingyuan/E664/2017 (H7N9-E664) (3), and A/Chicken/Guangdong/E157/2017 (H7N9-E157) were used in this study. Influenza viruses were propagated in 10-day-old specific-pathogen-free (SPF) embryonated chicken eggs. The viral allantoic fluid was harvested from each embryo and clarified at 4,000×*g* centrifugation for 5 min. The clarified fluid was then ultracentrifuged at 30,000×*g* for 1 h, and the virus solution was further purified using a 20%–30%–45%–60% discontinuous sucrose gradient. The 50% egg infectious dose (EID_50_) and the 50% egg lethal dose (ELD_50_) were calculated using the Reed-Muench method (30). Furthermore, H7N9-16876 and H7N9-E157 AIVs were used as challenge viruses. The inactivated virus using 0.1% formalin was used as hemagglutination inhibition (HI) antigen.

### Generation of Recombinant Baculovirus

To generate the VLP, the hemagglutinin (HA), neuraminidase (NA), and matrix protein (M1) genes derived from A/Chicken/Guangdong/16876/2016(H7N9) were biochemically synthesized by BGI (Shenzhen, China). Genes of HA, NA, and M1 were codon optimized for a high level of expression in High Five cells, then a 6xHis epitope tag was simultaneously fused to the C-terminal end of the optimized gene.

A recombinant chimeric protein containing honeybee melittin signal peptide, tandem repeat of 2HA_76-130_, 4M2e, 8NP_55-69_, and a flexible linker sequence (3xG4S) was designed and named HMN (**Table 1**). Each M2e sequence was linked by a linker sequence (PGGSSGGSS). Each NP_55-69_ sequence was linked by a linker sequence (GGSS), and the 6xHis tag epitope was linked to the 3′ ends of the HMN sequence by a GGSS linker. HMN gene was codon optimized for a high level of expression in the High Five cells and synthesized by BGI.

**Table 1:** Antigen epitopes included in HMN.

Epitope	Sequence
HA2 76-130	QIGNVINWTRDSITEVWSYNAELLVAMENQHTIDLADSE MDKLYERVKRQLRENA
M2e 2-24	SLLTEVETPTRTGWECNCSGSSD
NP 55-69	RLIQNSITIERMVLS
Melittin SP	MKFLVNVALVFMVVYISYIYAD

HA2 76–130, hemagglutinin stem area amino acids 76–130; M2e, the ectodomain of matrix protein M2; NP55–69, nucleoprotein amino acids 55–69; Melittin SP, melittin signal peptide.

These four optimized genes were cloned into the pACEBac1 vector plasmid (Invitrogen, Carlsbad, CA, USA), respectively. The recombinant plasmids were transformed into *Escherichia coli* DH10Bac to make recombinant bacmid baculovirus DNA, purified recombinant bacmid DNAs were transfected into sf9 insect cells using Cellfectin™ II reagent (Invitrogen) to obtain the recombinant baculovirus (rBV) in the culture supernatant. Following the manufacturer’s instructions, the recombinant baculoviruses were then amplified by infecting sf9 insect cells. All preparations of rBV were plaque purified and titrated using a rapid titration kit (BacPak Baculovirus Rapid Titer Kit; Clontech, Mountain View, CA, USA).

### Expression and Purification of H7N9-VLP and HMN

To generate recombinant proteins, High Five cells were maintained as suspension cultures in HF-SFM serum-free medium (World-Medium Biotechnology Co., Ltd., Suzhou, China) in shaker flasks at 27°C. For the production of VLP containing the H7N9 HA, NA, and M1 proteins, High Five cells were coinfected with rBVs expressing HA, NA, and M1, respectively, at a multiplicity of infection (MOI) of 2:1:2. After 3 days postinfection, cell culture supernatants were harvested by centrifugation at 2,000×*g* for 30 min at 4°C to remove debris. The VLPs in the supernatants were purified by ultracentrifugation at 30,000×*g* for 60 min at 4°C. The sedimented particles were resuspended in phosphate-buffered saline (PBS, pH 7.2) at 4°C overnight and further purified through a 20%–30%–45%–60% discontinuous sucrose gradient at 100,000×*g* for 1 h at 4°C (31). The functionality of HA protein incorporated into VLPs was quantified by hemagglutination assay (HA assay) using 1% (v/v) chicken red blood cells.

For the production of HMN proteins, High Five cells were infected with rBV expressing HMN protein in shaker flasks at an MOI of 1. After 3 days postinfection, the infected High Five cells were harvested and disrupted by ultrasonication for 30 min to prepare cell lysates under the condition of maintaining the temperature at 0°C–4°C. The sonicated cell lysates were cleared by low-speed centrifugation (10,000×*g* for 3 min at 4°C) to remove cell debris. The target proteins were purified using Ni-chromatography and used for further studies. The concentration of the purified VLPs and HMN was quantified using the Pierce BCA Protein Assay Kit (Thermo Fisher Scientific, Waltham, MA, USA).

The indirect immunofluorescence assay (IFA) was performed to detect the expression of VLPs and HMN protein in infected sf9 insect cells. Briefly, sf9 insect cells were infected with recombinant baculovirus expressing H7N9 proteins or HMN, respectively. After incubation for 48 h, the cells were fixed with 80% precooled acetone at −20°C for 15 min and incubated with the primary chicken antiserum against H7N9 AIVs or anti-His-tag mouse monoclonal antibody at a dilution of 1:200, and then with the secondary fluorescein isothiocyanate (FITC)-conjugated goat anti-chicken IgG antibody (Invitrogen, Carlsbad, CA, USA) or rabbit anti-mouse IgG antibody (Sigma St. Louis, MO, USA). Fluorescent images were examined under an inverted fluorescence microscope (Nikon, Ti-S, Minato, Japan).

### SDS-PAGE and Western Blot

The H7N9 VLPs and HMN proteins were analyzed using SDS-PAGE and Western blot. Briefly, the protein samples were mixed with 5x SDS-PAGE loading buffer (Dingguo, Guangzhou, China) and boiled for 10 min, then separated by 10% Tris-Glycine gels, and stained using Coomassie Brilliant Blue (Dingguo, Guangzhou, China) for SDS-PAGE analysis. The protein bands were also transferred to nitrocellulose membranes (Bio-Rad, Guangzhou, China) for Western blot analysis. The membranes were blocked with 5% (W/V) skim milk in PBST [PBS containing 0.05% (v/v) Tween 20] overnight at 4°C. Membranes were subsequently incubated with an anti-His-tag mouse monoclonal antibody (1:5,000, v/v, BioWorld Technology, Nanjing, China) for 1 h at room temperature. The blots were then washed five times with PBST and incubated with a horseradish-peroxidase-conjugated goat anti-mouse IgG antibody (LI-COR, Lincoln, NE, USA) for 1 h at room temperature. Finally, the proteins were visualized by chemiluminescence (LI-COR Odyssey).

### Electron Microscopy

Sucrose gradient-purified VLP samples were adsorbed onto a carbon parlodion-coated copper grid for 2 min. Excess VLP suspension was removed by blotting with filter paper, and the grid was immediately stained with 1% phosphotungstic acid for 10 min. Excess stain was removed by filter paper, and the samples were examined using a transmission electron microscope (Talos L120C, FEI, Czech).

### Vaccination and Challenge

Three-week-old SPF chickens were purchased from the Experimental Animal Center (Xinxing Dahuanong Eggs Co., Ltd., Guangdong, China). They were maintained according to the South China Agricultural University’s guidelines for the care and use of laboratory animals and used to determine the immunogenicity and efficacy of the H7N9 VLPs. The commercial avian influenza trivalent inactivated vaccine [Reassortant Avian Influenza Virus (H5+H7) Trivalent Vaccine, Inactivated (H5N2 Strain rSD57+ Strain rFJ56, H7N9 Strain rLN79)] was provided from South China Biological Medicine Co., Ltd. (Guangzhou, China). For a homologous protection study, a group (*N* = 10) of chickens were subcutaneously immunized once with 30 μg of (total protein) VLPs in combination with EOLANE 150 (Total Energies, Paris, France). The commercial avian influenza trivalent inactivated vaccine (H7+H5) was set as comparison control, and one group of chickens was inoculated with PBS as a negative control. Three weeks after immunization, chickens were intranasally challenged with 2 × 10^6.0^ ELD_50_ (0.2 ml) HPAI H7N9-16876.

For a crossprotection study, groups (*N* = 13 each group) of chickens were subcutaneously immunized once with 30 μg of VLP with ISA 201 VG, ISA 201 VG supplemented with 30 μg of HMN, and ISA 201 VG supplemented with 30 μg of Poly(I:C) (*In vivo*Gen, San Diego, CA, USA); one group (*N* = 13) of chickens was immunized intramuscularly once with 30 μg of VLP with ISA 71VG (Seppic, Paris, France), and one group of chickens was inoculated PBS as a negative control. Nineteen days after immunization, the peripheral blood and spleen of chickens (*N* = 3) in each group were obtained for the determination of cytokine levels. Three weeks after immunization, other chickens (*N* = 10) of each test group were inoculated intranasally with 10^6.0^ EID_50_ of H7N9-E157 virus in a 200-μl volume. Chickens were monitored for clinical signs and mortality for 14 days postchallenge (PC). All surviving chickens were killed humanely at the end of monitoring experiments.

To determine virus positivity or shedding from individual chickens, the oropharyngeal and cloacal swab samples were collected at 5 days postchallenge in the homologous protection study. The oropharyngeal and cloacal swab samples were collected at 3, 5, 7, and 9 days postchallenge in the crossprotection study. The swab samples were resuspended in 1 ml of PBS supplemented with 2,000 mg/ml streptomycin and 2,000 IU/ml penicillin. The suspensions were centrifuged at 3,000×*g* for 10 min, and 0.1 ml of the supernatants from the oropharyngeal or cloacal swabs were used to inoculate the allantoic cavities of 10-day-old SPF chicken embryos (3 eggs/sample). After incubation for 48 h at 37°C, the allantoic fluids were tested for hemagglutination activity. A virus isolation positive swab means one or more of the inoculated egg allantoic fluids reciprocal to the hemagglutination titers was higher than 4.

### Serology Assays

To determine the immunogenicity of the vaccines, serum antibody levels were titrated by hemagglutination inhibition (HI) assay or neutralization assay. Hemagglutination inhibition (HI) assay was performed using standard methods (32). Briefly, sera were pretreated with a receptor destroying enzyme (RDE, Seiken, Japan) for 20 h at 37°C followed by inactivation of the RDE at 56°C for 30 min. Twofold serial dilutions of 50 µl pretreated sera were incubated with an equal volume of 4 HA units of the inactivated H7N9 virus antigen for 1 h at room temperature. Then, 50 µl of a 1% suspension of chicken red blood cells (RBC) was added to each well and incubated at room temperature for 30 min. The HI titer was expressed as the reciprocal of the highest serum dilution that completely inhibited hemagglutination of 4 HA units of the virus. The neutralization assay was performed as follows. Briefly, MDCK cells were plated into 96-well plates. The twofold serial dilutions of heat-inactivated (56°C, 30 min) serum samples were mixed with equal volumes of 100 mean tissue culture infective doses (TCID50) of H7N9 influenza viruses (E157 or E664). After 1 h of incubation at 37°C, the mixtures of serum and virus were added to the MDCK cells. Cells were then incubated for 1 h at 37°C. After 1 h of incubation, the culture supernatants were replaced by medium supplemented with 0.5 μg/μl TPCK-trypsin (Dingguo, Guangzhou, China), and cells were incubated for an additional 72 h. After 72 h of incubation, cell supernatants were harvested and transferred to V-bottom 96-well plates. The presence of virus was detected using a hemagglutination assay (33). Neutralizing antibody titers were defined as the reciprocal of the highest serum dilution that neutralized the virus in cell supernatants.

### Isolation and Stimulation of Chicken PBMCs and Splenocytes

Peripheral blood mononuclear cells (PBMCs) and splenocytes were prepared for cytokine assays. PBMCs were isolated from peripheral blood using Ficoll-Hypaque density sedimentation (Tbdscience, Tianjin, China). Splenocytes were obtained from the spleens of chickens by density gradient centrifugation using Lymphoprep (Tbdscience, Tianjin, China) according to the manufacturer’s instructions. After contaminating red blood cells (RBC) present in the isolated cells lysed using RBC lysis buffer (Solarbio, Beijing, China), single cells were collected. PBMCs and splenocyte single-cell suspensions were cultured in complete Roswell Park Memorial Institute (RPMI) 1640 medium containing 10% FBS and 1% penicillin-streptomycin/l-glutamine (Gibco, Carlsbad, CA, USA) at a final concentration of 1 × 10^6^ cells/ml. Cells were stimulated with 20 μg of inactivated H7N9-E157 virus or H7N9 VLPs and incubated for 8 h at 37°C. Cells were then harvested for RNA extraction. Cytokine expression levels of cells were evaluated using qRT-PCR.

### Cytokine Assays Using Quantitative Real-Time PCR (qRT-PCR)

Total mRNA was extracted using total RNA extraction kits (Feijie, Shanghai, China); 500 ng of total mRNA was converted into cDNA using HiScript Reverse Transcriptase (Vazyme, Nanjing, China) according to the manufacturer’s instructions. mRNA expressions were examined using quantitative real-time PCR (qRT-PCR) with ChamQ Universal SYBR qPCR master mix (Vazyme, Nanjing, China) using a Bio-Rad CFX Applied System PCR instrument (Bio-Rad Laboratories Inc., Hercules, CA, USA). Sequences of primers used for qRT-PCR are shown in **Table 2**. The analyzed specific gene level was normalized with a housekeeping gene β-actin of the respective treatment group, and results were expressed in fold change.

**Table 2:** Sequences of primers used for quantitative real-time PCR.

Gene	Primer Sequences (5′–3′)	Product Size (bp)	Accession No.
IFN-γ	F: ACCTTCCTGATGGCGTGAAG	102	AJ634956.1
	R: TGAAGAGTTCATTCGCGGCT		
IL-4	F: ATGACATCCAGGGAGAGGTTT	235	GU119892.1
	R: ATTGGAGTAGTGTTGCCTGCT		
IL-17	F: ACAGGAGATCCTCGTCCTCC	95	AY744450.1
	R: TGACACATGTGCAGCCCAC		
β-Actin	F: TGGGTATGGAGTCCTGTGGT	136	NM_205518.1
	R: CTGTCAGCAATGCCAGGGTA		

### Statistical Analysis

Experimental data are presented as mean ± SD of the mean. GraphPad Prism 7 software was used for data analysis. The results of serum antibody titers and cytokine level were evaluated using one-way ANOVA and Tukey’s multiple-comparison test. Significant differences are denoted by an asterisk as follows: ^*^ *p* < 0.05, ^**^ *p* < 0.01, ^***^ *p* < 0.001, or ^****^ *p* < 0.0001.

## Results

### Production and Characterization of H7N9 VLPs and HMN

**Figure 1:**
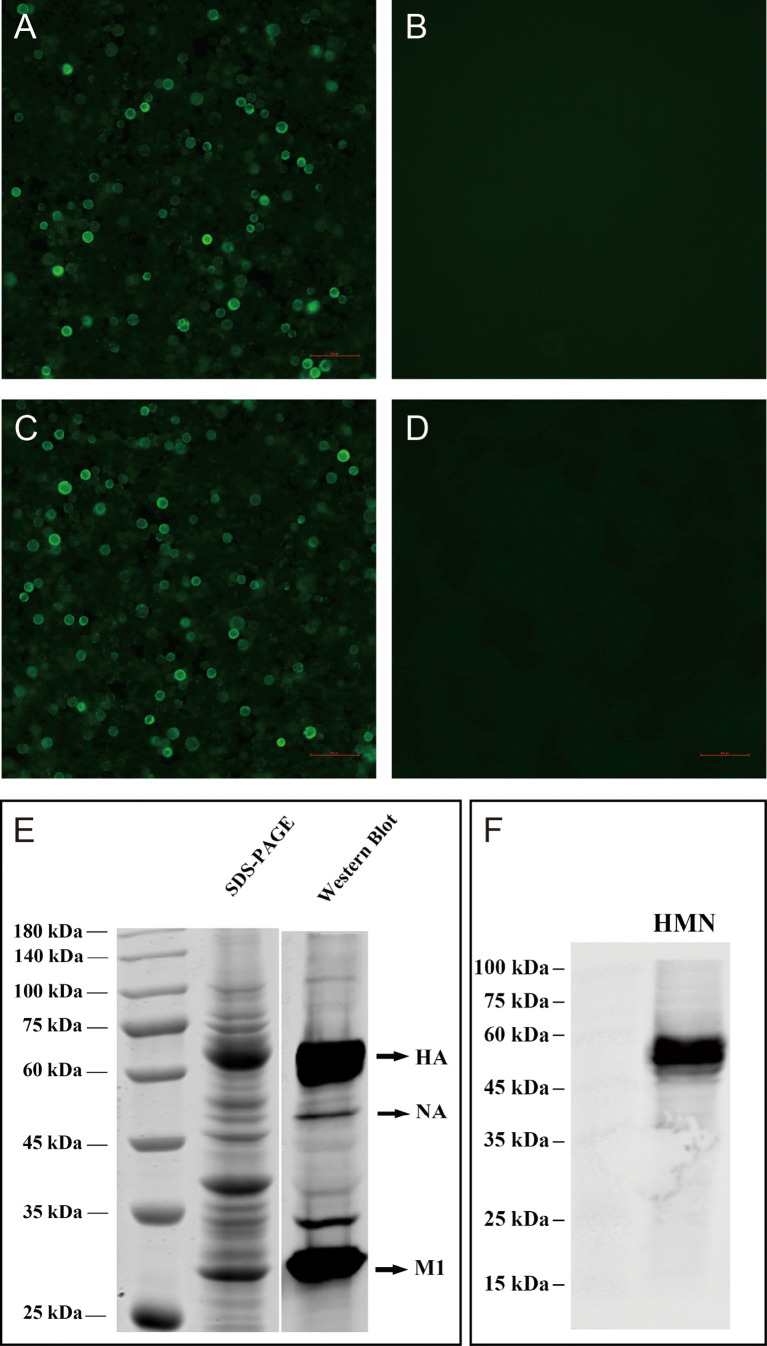
Characterization of H7N9 VLPs and HMN by indirect immunofluorescence assay (IFA), SDS-PAGE, and Western blotting. IFA detection of expression of the baculovirus in sf9 infected cells. Sf9 cells infected with rBV-HA, rBV-NA, and rBV-HA **(A)**, rBV-HMN **(C)**, or only empty baculoviruses **(B)**, **(D)** after 48 h. H7N9 chicken antiserum and anti-His-tag mouse monoclonal antibodies were used in the IFA assay. **(E)** The expression of the HA, NA, and M1 proteins on the VLPs was analyzed using SDS-PAGE gels with Coomassie blue staining and validated by Western blot using the anti-His-tag mouse monoclonal antibody. The molecular mass of H7N9 HA, NA, and M1 were ~70, ~55, and ~28 kDa, respectively. **(F)** The expression of HMN protein was validated by Western blot using the anti-His-tag mouse monoclonal antibody. The molecular mass of HMN was ~52 kDa.

**Figure 2:**
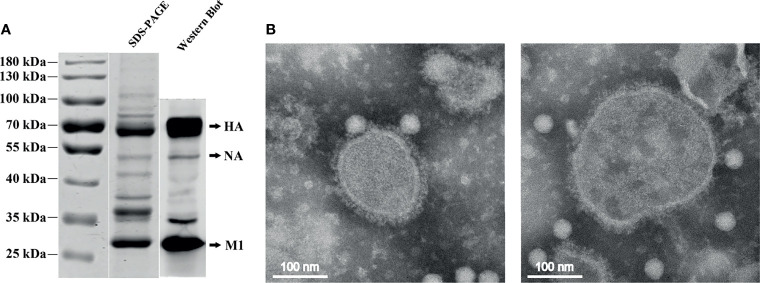
SDS-PAGE, Western blotting, and electron microscopy of the purified H7N9 VLPs. **(A)** The purity of the purified H7N9 VLPs was analyzed using SDS-PAGE and validated by Western blotting with the anti-His-tag mouse monoclonal antibody. **(B)** Negative staining electron microscopy of the H7N9 VLPs. Purified VLPs were stained using 1% phosphotungstic acid.

The H7N9 VLPs were produced in High Five insect cells, which were coinfected with recombinant baculovirus (rBVs) expressing HA, NA, and M1. Based on optimization results, the H7N9 VLPs in the study were produced using High Five cells coinfected with rBVs expressing HA, NA, and M1 at an MOI of 2:1:2. The expression of H7N9 proteins was observed with indirect IFA with chicken antiserum against H7N9 AIVs in sf9 cells 48 h after coinfection with HA, NA, and M1 rBVs (**Figure 1A**), whereas there was no specific fluorescence in the control baculovirus-infected cells (**Figure 1B**). The production of VLPs from cell culture supernatants was confirmed using SDS-PAGE and Western blotting (**Figure 1E**). The molecular mass of HA, NA, and M1 proteins was ~70, ~53, and ~28 kDa, respectively. The VLPs were then purified using the sucrose gradient centrifugation. The purity of the H7N9 VLPs was confirmed using SDS-PAGE and Western blotting (**Figure 2A**). The hemagglutination activity of the purified H7N9 VLPs reached 2^13^. The size and morphology of H7N9 VLPs were examined by transmission electron microscopy (**Figure 2B**). The average size of the VLPs was 100 nm; the morphology of the VLPs resembles that of influenza virus particles, and the spikes were observed on spherical surfaces which mimic influenza virus HA and NA proteins on the native virions.

**Figure 3:**
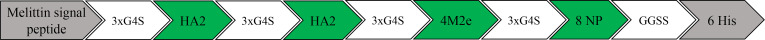
Schematic representation of HMN structure. HMN: melittin signal peptide; two repeated copies of the hemagglutinin stem area amino acids 76-130 (2HA2); four repeated copies of the ectodomain of matrix protein M2 (4M2e); eight repeated copies of the nucleoprotein amino acids 55-69 (8NP); 6xHis tag epitope. 3xG4S, GGGGSGGGGSGGGGS.

The HMN fusion construct was generated as described in **Figure 3**. HMN gene consists of the 2HA2_76-130_, 4M2e, and 8NP_55–69_ epitope sequences, a linker sequence, melittin signal peptide, and 6xHis tag epitope. The expression of HMN proteins was observed using an IFA in HMN rBV-infected sf9 cells (**Figure 1C**), whereas there was no specific fluorescence in control baculovirus-infected cells (**Figure 1D**). Western blot analysis was used to validate HMN protein (**Figure 1F**). The determined molecular mass of the HMN protein was ~52 kDa.

### H7N9 VLP Vaccines Elicit Immune Responses in Chickens

**Figure 4:**
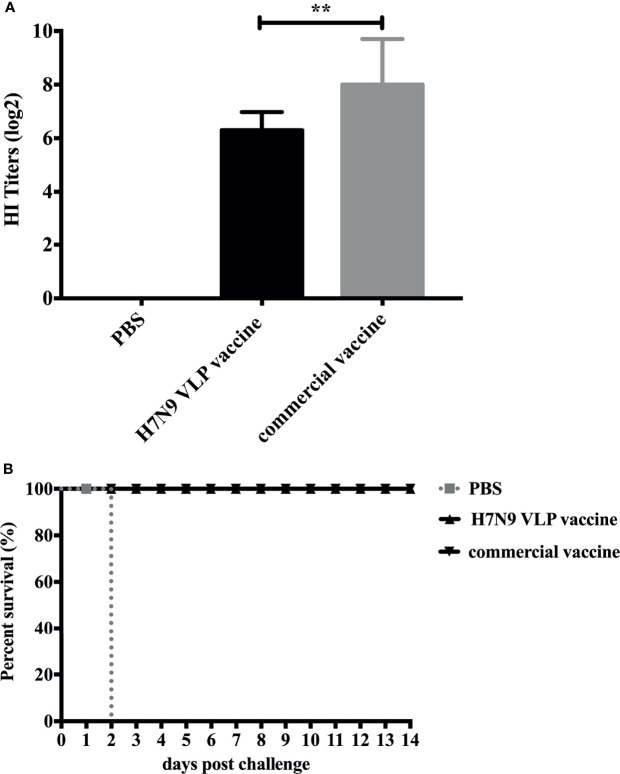
Hemagglutinin inhibition (HI) titers of SPF chickens after immunization and survival rates of SPF chickens after challenge. **(A)** The SPF chickens were immunized with the H7N9 VLP vaccine and the H7N9 commercial vaccine. The serum samples were collected at 3 weeks postvaccination to measure the HI antibody titers. The HI titers of SPF chicken sera were measured with 4 HAU testing antigens of the H7N9 GD16 virus. The HI titers among vaccination groups were compared using one-way ANOVA followed by Tukey’s multiple-comparison test. ^**^ *p* < 0.01, statistically significant differences. **(B)** At 3 weeks postvaccination, groups of SPF chickens (*n* = 10) were intranasally challenged with a high lethal dose (2 × 10^6.0^ ELD_50_) of A/Chicken/Guangdong/GD16/2016 H7N9 AIVs. Survival rates of chickens were measured daily for 2 weeks after challenge.

To examine the capacity of H7N9 VLP vaccine to induce immune responses in chickens, groups of 3-week-old SPF chickens were subcutaneously vaccinated one time with 30 μg of H7N9 VLPs formulated with adjuvant EOLANE 150 and H7N9 commercial vaccine as controls. The level of serum antibody against homologous virus H7N9-16876 was measured by HI assay at 3 weeks after a single-dose vaccination. The result showed that all vaccine groups effectively elicited anti-H7N9 AIV HI antibodies; the HI titers of chickens receiving the H7N9 commercial vaccine were higher than those induced by receiving the H7N9 VLP vaccine (**Figure 4A**). The mean HI titers of the H7N9 VLP vaccine reached 6.5 log_2_, which showed that the H7N9 VLP vaccine induced a high antibody response in chickens.

### H7N9 VLP Vaccines Offer Protection Against a High Lethal Dose Challenge of Homologous H7N9 Virus

Groups of 3-week-old SPF chickens were subcutaneously vaccinated once with EOLANE 150-adjuvanted H7N9 VLP vaccine or H7N9 commercial vaccine, respectively; the control group was treated with PBS. All chickens were intranasally challenged with 2 × 10^6.0^ ELD_50_ (0.2 ml) of A/Chicken/Guangdong/16876/2016 (H7N9) virus 3 weeks after immunization. The survival rates and morbidity of chickens in each group were monitored for 2 weeks after the challenge. All chickens in the H7N9 VLPs and H7N9 commercial vaccine group survived the infection. In contrast, all chickens in the control group died of infection 2 days postchallenge (**Figure 4B**). The clinical signs of the vaccinated chickens were not observed, and the bodyweight still slightly increased in chickens that received the H7N9 VLPs and commercial vaccines during 14 days of the monitoring period (date not shown).

The excreted viruses *via* the oropharynx and cloaca were analyzed to determine the virus replication at 5 days postchallenge (**Table 3**). After the challenge, virus shedding was not detected in chickens from the H7N9 commercial vaccine group, and one chicken was positive for virus isolation in the H7N9 VLP vaccine group. Overall, although the HI titers induced by the H7N9 VLP vaccine were lower than those by the commercial vaccine, the protective efficacy of the H7N9 VLP vaccine was comparable with the commercial vaccine.

**Table 3:** Virus shedding after a lethal-dose homologous influenza virus challenge of chickens.

Group	Challenge Virus	5 dpc		Total Virus Shedding/total	No. Clinical Symptoms	Survival/Total
		Oropharyngeal Swab	Cloacal Swab			
H7N9 VLP vaccine	16876	1/10	0/10	1/10	0	10/10
Commercial vaccine	16876	0/10	0/10	0/10	0	10/10
PBS	16876	NA	NA	NA	10	0/10

16876 is virus of A/Chicken/Guangdong/16876/2016 (H7N9). The oropharyngeal and cloacal swab samples were collected at 5 days postchallenge. Virus positivity or shedding was determined by inoculating each swab solution into 3 eggs of 10-day-old specific-pathogen-free chicken embryos.

dpc, days postchallenge; NA, not applicable due to death of chickens.

### H7N9 VLP Vaccines Induce Crossreactive HI and Neutralizing Antibody Against Antigenically Divergent H7N9 Viruses

**Figure 5:**
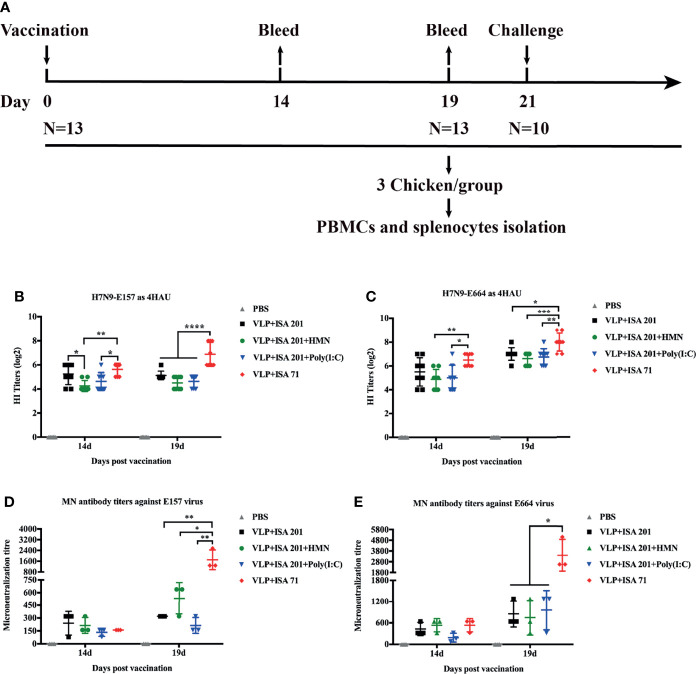
Hemagglutinin inhibition (HI) and neutralizing antibody titers of immune sera from vaccinated specific pathogen-free (SPF) chickens. **(A)** The timeline and vaccination and challenge study in chicken. SPF chickens were immunized with H7N9 VLP vaccine candidates, and the serum samples were collected 14 and 19 days after immunization. The HI titers of SPF chicken sera were measured with 4 HAU of E157 **(B)** and E664 **(C)**. Viral neutralizing antibody titers in serum were determined with E157 **(D)** and E664 **(E)** AIVs. The neutralizing and HI antibody titers among vaccination groups were compared using one-way ANOVA followed by Tukey’s multiple-comparison test. Statistically significant differences are indicated by an asterisk as follows: ^*^ *p* < 0.05, ^**^ *p* < 0.01, ^***^ *p* < 0.001, or ^****^ *p* < 0.0001.

To evaluate the crossreactivity of the serum antibodies from the H7N9 VLP-vaccinated chickens against antigenically divergent H7N9 AIVs from wave 5, HI and neutralization assay were carried out against H7N9 variant viruses E157 and E664 (3). Groups of 3-week-old SPF chickens were immunized once with 30 μg of H7N9 VLPs formulated with ISA 201 VG, ISA 201 VG plus HMN (30 μg), ISA 201 VG plus Poly(I:C) (30 μg), and ISA 71 VG. Antisera were collected at 14 and 19 days after a single-dose vaccination (**Figure 5A**). For HI assay, using 4 HA units (HAU) of H7N9-E157 as a testing virus, the results showed that ISA 71 VG-adjuvanted H7N9 VLP vaccine immunization could induce a higher level of HI antibody titers, which was significantly higher than that induced by the ISA 201 VG-containing adjuvant H7N9 VLP vaccine. Furthermore, the use of ISA 201 VG adjuvant alone showed slightly higher HI titers than the titer observed with ISA 201 VG plus HMN and ISA 201 VG plus Poly(I:C), but the difference was not statistically significant at 3 weeks after immunization (**Figure 5B**). Using 4 HAU of H7N9-E664 as a testing virus, the results showed the mean HI titers of the ISA 71 VG adjuvant group were 6.5 log_2_ 14 days after immunization, which was significantly higher than the titers of other adjuvant groups. The serum HI levels of all vaccine groups 19 days after immunization were substantially increased compared with those on day 14. The ISA 71 VG adjuvant group demonstrated significantly higher HI titers than the ISA 201 VG-containing adjuvant groups. There were no significant differences among ISA 201 VG-associated vaccine groups (**Figure 5C**).

Neutralization assay was carried out against the H7N9 AIV E157 or E664. Serum samples from the VLP+ISA 71 vaccine group demonstrated significantly higher neutralizing antibody titers than those from other vaccine groups (**Figures 5D, E**).

### Q226 Mutation on H7N9 Influenza Virus Hemagglutinin May Lead to Biased Antigenicity Evaluation

**Figure 6:**
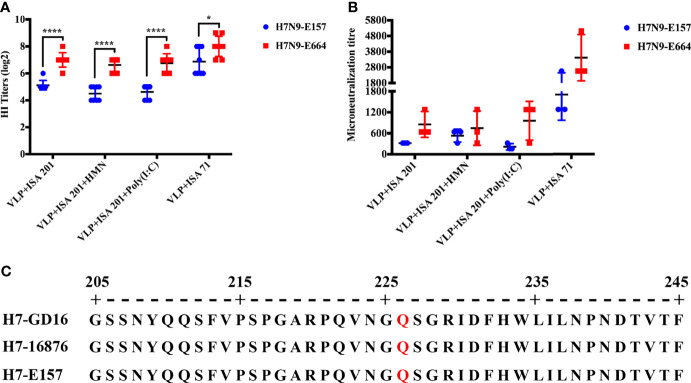
Q226 mutation of H7 HA decreases readouts of HI and neutralizing antibody titers. **(A)** HI titers of H7N9-VLP immune sera from chickens to H7N9-E157 and H7N9-E664 viruses. **(B)** Neutralizing antibody titers of H7N9-VLP immune sera from chickens to H7N9-E157 and H7N9-E664 viruses. **(C)** Sequence alignment of HA gene of the H7N9-GD16, H7N9-E157, and H7N9-E664 viruses. H7N9-GD16, A/Guangdong/17SF003/2016 (H7/GD16). Statistically significant differences are indicated by an asterisk as follows: ^*^ *p* < 0.05, ^****^ *p* < 0.0001.

The study has shown that the Q226 mutation in the HA of H7N9 influenza virus [A/Guangdong/17SF003/2016 (H7/GD16)] from the fifth wave increases the viral receptor-binding avidity to RBC, leading to decreasing HI titers against viruses containing HA Q226 and resulting in a biased antigenic evaluation based on HI assay (34). In this study, the fifth wave of H7N9 influenza virus E157 and E664 were used to evaluate the crossreactivity of H7N9 VLP vaccine sera. Nineteen days after immunization, the H7N9-E157 virus displayed significantly lower HI titers to H7N9-VLP immune sera from all vaccine groups than the H7N9-E664 virus (**Figure 6A**). Similar results were observed in the neutralizing antibody titers (**Figure 6B**). By aligning the amino acid sequence of the HA gene of H7N9-GD16, H7N9-16876, and H7N9-E157 viruses, the results showed that the receptor-binding site of the HA gene of H7N9-16876 and H7N9-E157 viruses has the same Q226 mutation as that of the H7N9-GD16 virus (**Figure 6C**). These results showed that the Q226 mutation in the receptor-binding site of H7 HA decreased readouts of HI and neutralizing antibody titers by impacting the receptor-binding avidity to red blood cells.

### HMN and Poly(I:C) Enhance Th1-Type Immune Responses of H7N9 VLP Vaccine

**Figure 7:**
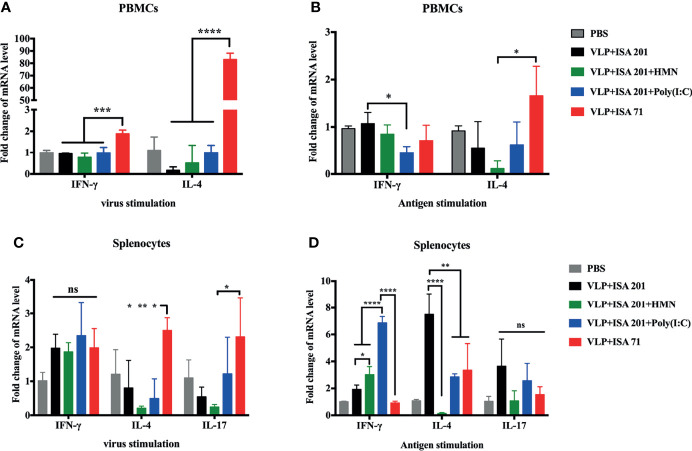
Cytokine expression levels in PBMCs and splenocytes of SPF chickens (N=3). PBMCs and splenocytes were isolated from the vaccinated chickens 19 days after immunization and stimulated with inactivated influenza virus or purified H7N9 VLPs antigen for 8 h in vitro. The expression levels of cytokines in PBMCs **(A, B)** and splenocytes **(C, D)** were measured using qRT-PCR. Data are presented as the mean ± standard error of the mean. Statistical significance of differences is illustrated as follows: *P < 0.05, **P < 0.01, ***P < 0.001, ****P < 0.0001, or ns, not significant.

To evaluate the ability of vaccine candidates to induce immune responses and to further estimate the immune types, PBMCs and splenocytes were isolated from the vaccinated chickens 19 days after immunization and stimulated with inactivated influenza virus or purified H7N9 VLP antigen *in vitro*. The level of the cytokines IFN-γ, IL-4, and IL-17, associated with Th1-type, Th2-type, and Th17-type immune responses, respectively, were determined to evaluate the immune types induced by H7N9 VLP vaccine candidates. After virus stimulation *in vitro*, mRNA expression levels of IFN-γ and IL-4 were significantly higher in the PBMCs of chickens that received ISA 71 VG-adjuvanted vaccine than levels of IFN-γ and IL-4 in the PBMCs of chickens that received ISA 201 VG-containing adjuvanted vaccine. The mRNA levels of IFN-γ were significantly higher in the PBMCs of VLPs in combination with ISA 71 VG-vaccinated chickens than that of the IL-4, indicating that Th2-biased immune responses were induced by ISA 71 VG-adjuvanted vaccine (**Figure 7A**). The splenocytes of chickens that received the ISA 71 VG-adjuvanted vaccine demonstrated significantly higher IL-4 and IL-17 mRNA expression levels than those immunized with the ISA 201 VG-containing adjuvant vaccine (**Figure 7C**). After antigen stimulation *in vitro*, the lowest levels of IFN-γ and IL-4 were observed in the PBMCs of chickens that received ISA 201 VG-adjuvanted vaccine in the presence of Poly(I:C) or HMN, respectively (**Figure 7B**). In contrast, the splenocytes of chickens that received Poly(I:C)-supplemented ISA 201 VG-adjuvanted vaccine could induce the highest expression levels of IFN-γ. mRNA levels of IFN-γ were significantly higher in the splenocytes of chickens vaccinated with HMN- or Poly(I:C)-supplemented ISA 201 VG-adjuvanted vaccine than with ISA 201 VG-adjuvanted vaccine alone. The splenocytes of chickens that received the ISA 201 VG-adjuvanted vaccine showed the highest IL-4 mRNA expression levels, which was significantly higher than the IL-4 levels in the splenocytes of chickens that received other H7N9 vaccine candidates (**Figure 7D**). The results indicated that the ISA 201 VG adjuvant vaccine supplement with Poly(I:C) or HMN induced Th1-biased immune responses. The use of ISA 201 VG or ISA 71 VG adjuvant alone induced Th2-biased immune responses.

### H7N9 VLP Vaccine Confers Crossprotection Against Heterologous H7N9 Virus

**Figure 8:**
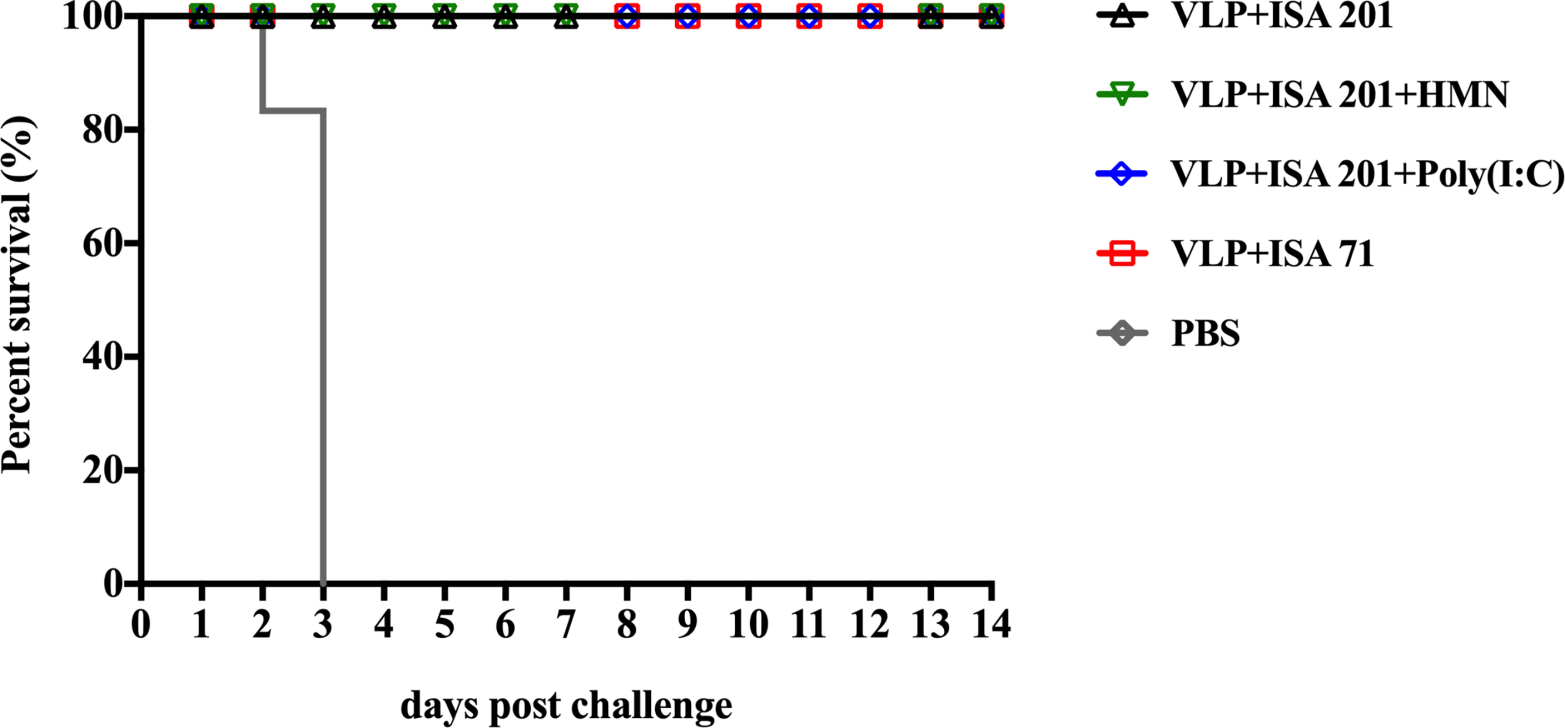
Survival rates of the specific pathogen-free (SPF) chickens after H7N9-E157 virus challenge. At 3 weeks postvaccination, groups of SPF chickens (*n* = 10) were intranasally challenged with a high lethal dose (10^6.0^ EID_50_) of A/Chicken/Guangdong/E157/2017 H7N9 AIVs. Survival rates of chickens were measured daily for 2 weeks after challenge.

Prior to challenge, the results showed that the titers of HI and neutralizing antibody titers against H7N9-E157 AIVs were lower than those against H7N9-E664 AIVs. Therefore, to effectively evaluate the crossprotective efficacy of the H7N9 VLP vaccine, the H7N9-E157 AIVs were selected as challenge virus. Groups of 3-week-old SPF chickens were vaccinated once with VLP+ISA 201, VLP+ISA 201+HMN, VLP+ISA 201+Poly(I:C), and VLP+ISA 71 vaccines, respectively; the control group was treated with PBS. All chickens were intranasally challenged with 10^6.0^ EID_50_ (0.2 ml) of the H7N9-E157 virus 3 weeks after immunization. All chickens in the vaccine groups of VLP+ISA 201, VLP+ISA 201+HMN, VLP+ISA 201+Poly(I:C), and VLP+ISA 71 survived the infection. In contrast, all chickens in the control group died of infection 3 days PC (**Figure 8**). The clinical signs of the vaccinated chickens were not observed, and the bodyweight still slightly increased in chickens that received the H7N9 VLP vaccine candidates during 14 days of the monitoring period (data not shown).

The excreted viruses *via* the oropharynx and cloaca were analyzed to determine the virus replication at 3, 5, 7, and 9 days PC (**Table 4**). After the challenge, virus shedding was not detected in chickens from the VLP+ISA 201+HMN group, and one chicken in the VLP+ISA 201+ Poly(I:C) group, two chickens in the VLP+ISA 201 group, and one chicken in the VLP+ISA 71 group recovered viruses from cloacal swab samples.

**Table 4:** Virus shedding after a lethal-dose E157 AIV challenge of chickens.

Group	Oropharyngeal swab (virus shedding number/total number)				Cloacal swab (virus shedding number/total number)				No. clinical symptoms
	3 dpc	5 dpc	7 dpc	9 dpc	3 dpc	5 dpc	7 dpc	9 dpc	
VLP+ISA 201	0/10	0/10	0/10	0/10	0/10	2/10	0/10	0/10	0
VLP+ISA 201+HMN	0/10	0/10	0/10	0/10	0/10	0/10	0/10	0/10	0
VLP+ISA 201+Poly(I:C)	0/10	0/10	0/10	0/10	0/10	1/10	0/10	0/10	0
VLP+ISA 71	0/10	0/10	0/10	0/10	0/10	0/10	0/10	1/10	0
PBS	NA	NA	NA	NA	NA	NA	NA	NA	10

E157 is virus of A/Chicken/Guangdong/E157/2017. The oropharyngeal and cloacal swab samples were collected at 3, 5, 7, and 9 days postchallenge. Virus positivity or shedding was determined by inoculating each swab solution into 3 eggs of 10-day-old specific-pathogen-free chicken embryos.

dpc, days postchallenge; NA, not applicable due to death of chickens.

## Discussion

The continuous evolution and mutation of the H7N9 avian influenza virus poses a dual threat to human health and the poultry industry (4), and thus it is imperative to develop a safe and effective vaccine against H7N9 virus infections. A variety of the influenza vaccine formations were designed to protect against influenza virus (35–38). Avian influenza VLPs retain the structural and antigenic properties of native viruses but lack the genetic material, which is a better selection as vaccine antigen for the development of influenza vaccine. Influenza VLPs have been generated in various production platforms, including plant cells (39, 40), hepatitis B virus core (HBc) (41, 42), insect cells (11, 43), and mammalian cells (44). Commercially, the method for producing influenza VLPs using a baculovirus expression vector system (BEVS) is safe and low cost. High-throughput production of avian influenza VLPs is possible by optimizing the BEVS process (12, 45). A previous study showed that intramuscular and intranasal immunization with insect cell-derived H7 VLPs induces protective immunity against lethal H7N9 virus challenge (13), which supports the hypothesis that influenza VLPs are a promising candidate H7N9 vaccine antigen.

HA and NA proteins are the major surface glycoproteins of influenza viruses. Reports have shown that the recombinant H7 HA subunit vaccine protects mice from H7N9 influenza virus challenge (16, 17). NA expressing VLPs induced effective crossprotective immunity against influenza virus (46). On the other hand, M1 is a central component of the virus particle. In this study, we developed VLPs containing H7N9 HA, NA, and M1 proteins. The expression of VLP was detected using SDS-PAGE, and VLP antigenicity was validated *via* IFA and Western blotting. The hemagglutination activity of VLPs further confirmed that HA proteins anchored on surface VLPs retained functional stability and cRBC-binding activity. The water-in-oil-in-water emulsion adjuvant Montanide ISA 201 VG was used as an adjuvant in conjunction with VLPs to improve the crossprotection of vaccination. Report has shown that Montanide ISA 201 VG combined with inactivated influenza virus induces both humoral and cell-mediated immune responses to protect against a homologous H1N1 challenge in swine (47). We demonstrated that vaccination with H7 VLPs combined with ISA 201 VG induced strong HI antibody titers after single-dose vaccination and homologous protection against H7N9-16876 virus challenge in chickens. However, the influenza VLP vaccine induced a lower HI antibody titer and presented virus shedding in vaccinated chickens compared with the commercial H7N9 inactivated vaccine. A previous study also showed that H7N9 VLPs combined with ISA 71 R adjuvant induced lower levels of HI and MN antibody titers compared with commercial whole-virus inactivated vaccine (6). The protective efficacy of the H7N9 VLPs vaccine can be further improved by increasing the dose of VLP (13). Previous studies have shown that recombinant baculovirus vaccine expressing H7N9 HA protein confers better protection to the inactivated vaccine in chickens (48), and a plant-derived H6N2 VLP elicited a better protective immunity than a commercial inactivated H6N2 vaccine (49). Therefore, the efficacy of the H7N9 VLP vaccine needs to be further evaluated.

In the present study, we developed a series of vaccination strategies for VLP vaccine aiming to enhance crossprotection and eliminate viral shedding in vaccinated chickens. Previous studies have shown that influenza conserved fusion epitopes vaccine can induce broad crossprotection against different influenza viruses (50, 51). However, the influenza vaccine based on influenza conserved epitopes cannot provide complete protection against influenza virus challenge in the presence of morbidity, mortality, and virus shedding (52–54). Therefore, a single recombinant protein based on influenza conserved epitopes is not sufficient as an influenza vaccine antigen. Reports have shown that supplementing influenza vaccines with tandem repeat M2e VLPs enhances crossprotection against homologous and heterologous influenza virus challenge in an animal model (55, 56). Therefore, the influenza conserved epitopes can be used as an antigen supplement to enhance the crossprotection ability of influenza VLP vaccines. In this study, we constructed and expressed a recombinant protein (HMN) based on influenza conserved peptides to improve the crossprotective immunity of influenza VLP vaccines. HMN protein antigenicity was validated *via* Western blotting. In this study, we demonstrated that supplementation of the VLP+ISA 201 vaccine with HMN protein cannot increase HI and neutralizing antibody titers to the VLP+ISA 201 vaccine. Nevertheless, following the heterologous H7N9-E157 virus challenge, viral shedding was completely abolished in chickens of the HMN supplement vaccine group, but two chickens in the VLP+ISA 201 group recovered viruses. Meanwhile, the HMN-supplemented VLP+ISA 201 vaccine induces significantly higher IFN-γ mRNA expression levels in splenocytes than the VLP+ISA 201 vaccine, which enhances the Th1-type immune responses of VLP+ISA 201 vaccine. A previous study showed that Th1 immune responses play a critical role in crossprotective immunity and virus removal against influenza virus challenge (57). Although this study is limited to not being able to determine the immune response induced by HMN, we speculate that the HMN protein may induce a broad crossprotective immune response and play an important role in virus clearance. Therefore, HMN protein as an antigen supplement is a promising vaccination strategy for crossprotection against the H7N9 influenza virus.

Influenza-specific cell-mediated immune responses play an important role in eliminating the virus in chickens receiving the influenza VLP vaccine (6). Poly(I:C) was selected as an adjuvant supplement to improve cell-mediated immune responses of the VLP+ISA 201 vaccine. Previous studies have shown that Poly(I:C)-adjuvanted influenza vaccines induce a cell-mediated immune response, conferring protection against homologous and heterologous virus challenge (28, 58). In this study, we demonstrated that the Poly(I:C)-supplemented vaccine stimulates the highest mRNA expression levels of IFN-γ in splenocytes that enhanced a Th1-mediated immune response of the VLP + ISA 201 vaccine and provided a crossprotection against a heterologous H7N9 E157 virus challenge in chickens. However, the supplementation of Poly(I:C) did not significantly increase HI and MN antibody titers and did not completely inhibit virus shedding in vaccinated chickens, which may be related to the injection route of Poly(I:C). Reports have shown that intranasal immunization with Poly(I:C)-adjuvanted influenza vaccines induces robust mucosal, humoral, and cellular immunity to protect against homologous and heterologous influenza virus challenge (28, 58, 59). The potency of VLPs with Poly(I:C) needs to be further investigated in chickens administered intranasally.

Montanide ISA 71 VG was used as an adjuvant for comparison with Montanide ISA 201 VG. Montanide ISA 71 VG is a commercial water-in-oil emulsion adjuvant and has been confirmed to stimulate both humoral and cellular immune responses (60). Recent studies have shown that combining influenza VLPs with ISA 71 VG induces protective immunity against lethal homologous virus challenge (6, 61). These findings indicated that ISA 71 VG is a promising VLP subunit vaccine adjuvant. This study has shown that H7N9 VLPs combined with ISA 71 VG induce higher titers of HI and MN antibody against heterologous H7N9 virus than the ISA 201 VG-adjuvanted vaccine. Meanwhile, ISA 71 VG stimulated significantly higher IFN-γ, IL-4, and IL-17 mRNA expression levels in PBMCs and splenocytes than stimulated by ISA 201 VG. Following the H7N9-E157 virus challenge, the ISA 71 VG vaccine group showed less virus shedding than the ISA 201 VG vaccine group. In comparison, the ISA 201 VG-adjuvanted VLP vaccine induced lower serum HI and MN antibody titers against the heterologous H7N9-E157 and H7N9-E664 viruses and Th2-biased immune responses in chickens. Following the E157 virus challenge, the VLP+ISA 201 vaccine did not eliminate virus shedding in chickens. However, supplementing the VLP+ISA 201 vaccine with HMN protein can achieve the goal of virus clearance in chickens. This study demonstrated that Q226 mutation in the receptor-binding site of H7 HA plays a crucial role in reducing the readouts of HI and neutralizing antibody titers by impacting the receptor-binding avidity to red blood cells. Therefore, viral receptor-binding avidity should be considered in evaluating an H7N9 candidate vaccine.

In the future, H7N9 VLP from this study may be further modified by combining with mucosal or nanoparticle adjuvant. Mucosal immune responses play an important role in defense against influenza virus infection. Several studies showed that the intranasal administration of influenza vaccine combined with the mucosal adjuvant induced crossprotection against divergent influenza subtypes (62, 63). Influenza nanoparticle vaccine is one of the strategies for developing a universal influenza vaccine. Previous studies showed that influenza nanoparticles induce broad protection against heterosubtypic influenza viruses (64, 65).

In summary, our results indicate that H7N9 VLP vaccine candidates induce a crossreactive serum immune response and provide effective crossprotection against homologous and heterologous H7N9 influenza viruse challenge. In addition, we successfully developed a combo vaccine consisting of H7N9 VLP and polyepitope HMN that confers full protection against antigenically divergent H7N9 virus challenge. Our results collectively suggest that the supplementation of the H7N9 VLP vaccine with polyepitope antigen will be a promising strategy for broad protection against an antigenically divergent H7N9 virus.

## Data Availability Statement

The raw data supporting the conclusions of this article will be made available by the authors, without undue reservation.

## Ethics Statement

All experiments involved in the live H7N9 avian influenza viruses (AIVs) were performed in a biosafety level 3 laboratory facilities at South China Agricultural University (SCAU) (CNAS BL0011) in accordance with protocols. All animals involved in the experiments were reviewed and approved by the Institutional Animal Care and Use Committee at SCAU and treated in accordance with the guidelines (2017A002).

## Author Contributions

HF, ML, and DK designed the research. DK, TC, XH, SL, and YG performed the experiments. DK, TC, and CJ analyzed the data. DK, ML, and HF participated in writing the paper. All authors reviewed the manuscript.

## Funding

This work was supported by the Innovation Leading Team Program of Guangzhou City (202009020009) and the Key Research and Development Program of Guangdong Province (2019B020218004).

## Conflict of Interest

The authors declare that the research was conducted in the absence of any commercial or financial relationships that could be construed as a potential conflict of interest.

## Publisher’s Note

All claims expressed in this article are solely those of the authors and do not necessarily represent those of their affiliated organizations, or those of the publisher, the editors and the reviewers. Any product that may be evaluated in this article, or claim that may be made by its manufacturer, is not guaranteed or endorsed by the publisher.

## References

[1] Liu D Shi W Shi Y Wang D Xiao H Li W. Origin and Diversity of Novel Avian Influenza A H7N9 Viruses Causing Human Infection: Phylogenetic, Structural, and Coalescent Analyses. Lancet (2013) 381(9881):1926–32. doi: 10.1016/S0140-6736(13)60938-1 23643111

[2] Bao L Bi Y Wong G Qi W Li F Lv Q. Diverse Biological Characteristics and Varied Virulence of H7N9 From Wave 5. Emerg Microbes Infect (2019) 8(1):94–102. doi: 10.1080/22221751.2018.1560234 30866763 PMC6456849

[3] Wu Y Hu J Jin X Li X Wang J Zhang M. Accelerated Evolution of H7N9 Subtype Influenza Virus Under Vaccination Pressure. Virol Sin (2021) 36(5):1124-32. doi: 10.1007/s12250-021-00383-x PMC8112217 33974230

[4] Zhang J Ye H Li H Ma K Qiu W Chen Y. Evolution and Antigenic Drift of Influenza A (H7N9) Viruses, China, 2017-2019. Emerg Infect Dis (2020) 26(8):1906–11. doi: 10.3201/eid2608.200244 PMC7392412 32687047

[5] Maurice A Halasa N. Preparing for the 2019-2020 Influenza Season. Pediatr Transplant (2020) 24(1):e13645. doi: 10.1111/petr.13645 31885157

[6] Li J Li R Zhang Q Peng P Wang X Gu M. H7N9 Influenza Virus-Like Particle Based on BEVS Protects Chickens From Lethal Challenge With Highly Pathogenic H7N9 Avian Influenza Virus. Vet Microbiol (2021) 258:109106. doi: 10.1016/j.vetmic.2021.109106 34004568

[7] Erlewyn-Lajeunesse M Brathwaite N Lucas JSA Warner JO. Recommendations for the Administration of Influenza Vaccine in Children Allergic to Egg. BMJ (2009) 339:b3680. doi: 10.1136/bmj.b3680 19755545

[8] Parker L Wharton SA Martin SR Cross K Lin Y Liu Y. Effects of Egg-Adaptation on Receptor-Binding and Antigenic Properties of Recent Influenza A (H3N2) Vaccine Viruses. J Gen Virol (2016) 97(6):1333–44. doi: 10.1099/jgv.0.000457 PMC5394856 26974849

[9] Durous L Rosa-Calatrava M Petiot E. Advances in Influenza Virus-Like Particles Bioprocesses. Expert Rev Vaccines (2019) 18(12):1285–300. doi: 10.1080/14760584.2019.1704262 31829068

[10] Frietze KM Peabody DS Chackerian B. Engineering Virus-Like Particles as Vaccine Platforms. Curr Opin Virol (2016) 18:44–9. doi: 10.1016/j.coviro.2016.03.001 PMC4983494 27039982

[11] Huang D Chao Y-C Lv Z Jan J-T Yang Y-C Hsiao P-W. Comparison of Chicken Immune Responses After Inoculation With H5 Avian Influenza Virus-Like Particles Produced by Insect Cells or Pupae. J Vet Res (2021) 65(2):139–45. doi: 10.2478/jvetres-2021-0026 PMC8256473 34250297

[12] Lai C-C Cheng Y-C Chen P-W Lin T-H Tzeng T-T Lu C-C. Process Development for Pandemic Influenza VLP Vaccine Production Using a Baculovirus Expression System. J Biol Eng (2019) 13(1):78. doi: 10.1186/s13036-019-0206-z 31666806 PMC6813129

[13] Ren Z Zhao Y Liu J Ji X Meng L Wang T. Intramuscular and Intranasal Immunization With an H7N9 Influenza Virus-Like Particle Vaccine Protects Mice Against Lethal Influenza Virus Challenge. Int Immunopharmacol (2018) 58:109–16. doi: 10.1016/j.intimp.2017.12.020 29571081

[14] Steele KH Stone BJ Franklin KM Fath-Goodin A Zhang X Jiang H. Improving the Baculovirus Expression Vector System With Vankyrin-Enhanced Technology. Biotechnol Prog (2017) 33(6):1496–507. doi: 10.1002/btpr.2516 PMC5786172 28649776

[15] Kang H-J Chu K-B Yoon K-W Eom G-D Mao J Kim M-J. Neuraminidase in Virus-Like Particles Contributes to the Protection Against High Dose of Avian Influenza Virus Challenge Infection. Pathogens (2021) 10(10):1291. doi: 10.3390/pathogens10101291 34684240 PMC8537550

[16] Chen T-H Liu W-C Chen IC Liu C-C Huang M-H Jan J-T. Recombinant Hemagglutinin Produced From Chinese Hamster Ovary (CHO) Stable Cell Clones and a PELC/CpG Combination Adjuvant for H7N9 Subunit Vaccine Development. Vaccine (2019) 37(47):6933–41. doi: 10.1016/j.vaccine.2019.02.040 PMC7115541 31383491

[17] Liu B Shi P Wang T Zhao Y Lu S Li X. Recombinant H7 Hemagglutinin Expressed in Glycoengineered Pichia Pastoris Forms Nanoparticles That Protect Mice From Challenge With H7N9 Influenza Virus. Vaccine (2020) 38(50):7938–48. doi: 10.1016/j.vaccine.2020.10.061 33131935

[18] Bernasconi V Bernocchi B Ye L Lê MQ Omokanye A Carpentier R. Porous Nanoparticles With Self-Adjuvanting M2e-Fusion Protein and Recombinant Hemagglutinin Provide Strong and Broadly Protective Immunity Against Influenza Virus Infections. Front Immunol (2018) 9:2060. doi: 10.3389/fimmu.2018.02060 30271406 PMC6146233

[19] Bhide Y Dong W Gribonika I Voshart D Meijerhof T de Vries-Idema J. Cross-Protective Potential and Protection-Relevant Immune Mechanisms of Whole Inactivated Influenza Virus Vaccines Are Determined by Adjuvants and Route of Immunization. Front Immunol (2019) 10:646. doi: 10.3389/fimmu.2019.00646 30984200 PMC6450434

[20] Choi A Bouzya B Cortés Franco K-D Stadlbauer D Rajabhathor A Rouxel RN. Chimeric Hemagglutinin-Based Influenza Virus Vaccines Induce Protective Stalk-Specific Humoral Immunity and Cellular Responses in Mice. Immunohorizons (2019) 3(4):133–48. doi: 10.4049/immunohorizons.1900022 PMC6485968 31032479

[21] Kim Y-J Lee Y-T Kim M-C Lee Y-N Kim K-H Ko E-J. Cross-Protective Efficacy of Influenza Virus M2e Containing Virus-Like Particles Is Superior to Hemagglutinin Vaccines and Variable Depending on the Genetic Backgrounds of Mice. Front Immunol (2017) 8:1730:1730. doi: 10.3389/fimmu.2017.01730 29276514 PMC5727122

[22] Liu W-C Nachbagauer R Stadlbauer D Solórzano A Berlanda-Scorza F García-Sastre A. Sequential Immunization With Live-Attenuated Chimeric Hemagglutinin-Based Vaccines Confers Heterosubtypic Immunity Against Influenza A Viruses in a Preclinical Ferret Model. Front Immunol (2019) 10:756. doi: 10.3389/fimmu.2019.00756 31105689 PMC6499175

[23] McMahon M Asthagiri Arunkumar G Liu W-C Stadlbauer D Albrecht RA Pavot V. Vaccination With Viral Vectors Expressing Chimeric Hemagglutinin, NP and M1 Antigens Protects Ferrets Against Influenza Virus Challenge. Front Immunol (2019) 10:2005:2005. doi: 10.3389/fimmu.2019.02005 31497029 PMC6712942

[24] Liu J Ren Z Wang H Zhao Y Wilker PR Yu Z. Influenza Virus-Like Particles Composed of Conserved Influenza Proteins and GPI-Anchored CCL28/GM-CSF Fusion Proteins Enhance Protective Immunity Against Homologous and Heterologous Viruses. Int Immunopharmacol (2018) 63:119–28. doi: 10.1016/j.intimp.2018.07.011 30081250

[25] Song L Xiong D Song H Wu L Zhang M Kang X. Mucosal and Systemic Immune Responses to Influenza H7N9 Antigen HA1–2 Co-Delivered Intranasally With Flagellin or Polyethyleneimine in Mice and Chickens. Front Immunol (2017) 8:326. doi: 10.3389/fimmu.2017.00326 28424686 PMC5380672

[26] Zhang Z Zhang J Zhang J Li Q Miao P Liu J. Coimmunization With Recombinant Epitope-Expressing Baculovirus Enhances Protective Effects of Inactivated H5N1 Vaccine Against Heterologous Virus. Vet Microbiol (2017) 203:143–8. doi: 10.1016/j.vetmic.2017.03.004 28619136

[27] Patil V Renu S Feliciano-Ruiz N Han Y Ramesh A Schrock J. Intranasal Delivery of Inactivated Influenza Virus and Poly(I:C) Adsorbed Corn-Based Nanoparticle Vaccine Elicited Robust Antigen-Specific Cell-Mediated Immune Responses in Maternal Antibody Positive Nursery Pigs. Front Immunol (2020) 11:596964. doi: 10.3389/fimmu.2020.596964 33391267 PMC7772411

[28] Renu S Feliciano-Ruiz N Ghimire S Han Y Schrock J Dhakal S. Poly(I:C) Augments Inactivated Influenza Virus-Chitosan Nanovaccine Induced Cell Mediated Immune Response in Pigs Vaccinated Intranasally. Vet Microbiol (2020) 242:108611. doi: 10.1016/j.vetmic.2020.108611 32122615

[29] Dong J Chen P Wang Y Lv Y Xiao J Li Q. Evaluation of the Immune Response of a H7N9 Candidate Vaccine Virus Derived From the Fifth Wave A/Guangdong/17sf003/2016. Antiviral Res (2020) 177:104776. doi: 10.1016/j.antiviral.2020.104776 32201204

[30] Reed LJ Muench H. A Simple Method of Estimating fifty Per Cent Endpoints. Am J Epidemiol (1938) 27(3):493–7. doi: 10.1093/oxfordjournals.aje.a118408

[31] Quan FS Huang C Compans RW Kang SM. Virus-Like Particle Vaccine Induces Protective Immunity Against Homologous and Heterologous Strains of Influenza Virus. J Virol (2007) 81(7):3514–24. doi: 10.1128/JVI.02052-06 PMC1866067 17251294

[32] Pushko P Tumpey TM Bu F Knell J Robinson R Smith G. Influenza Virus-Like Particles Comprised of the HA, NA, and M1 Proteins of H9N2 Influenza Virus Induce Protective Immune Responses in BALB/c Mice. Vaccine (2005) 23(50):5751–9. doi: 10.1016/j.vaccine.2005.07.098 16143432

[33] Budimir N Huckriede A Meijerhof T Boon L Gostick E Price DA. Induction of Heterosubtypic Cross-Protection Against Influenza by a Whole Inactivated Virus Vaccine: The Role of Viral Membrane Fusion Activity. PloS One (2012) 7(1):e30898. doi: 10.1371/journal.pone.0030898 22303469 PMC3267744

[34] Wang Y Lv Y Niu X Dong J Feng P Li Q. L226Q Mutation on Influenza H7N9 Virus Hemagglutinin Increases Receptor-Binding Avidity and Leads to Biased Antigenicity Evaluation. J Virol (2020) 94(20):e00667–20. doi: 10.1128/JVI.00667-20 PMC7527056 32796071

[35] Asthagiri Arunkumar G McMahon M Pavot V Aramouni M Ioannou A Lambe T. Vaccination With Viral Vectors Expressing NP, M1 and Chimeric Hemagglutinin Induces Broad Protection Against Influenza Virus Challenge in Mice. Vaccine (2019) 37(37):5567–77. doi: 10.1016/j.vaccine.2019.07.095 PMC6717082 31399277

[36] Lu Y Landreth S Liu G Brownlie R Gaba A Littel-van den Hurk S. Innate Immunemodulator Containing Adjuvant Formulated HA Based Vaccine Protects Mice From Lethal Infection of Highly Pathogenic Avian Influenza H5N1 Virus. Vaccine (2020) 38(10):2387–95. doi: 10.1016/j.vaccine.2020.01.051 32014270

[37] Wang W Li R Deng Y Lu N Chen H Meng X. Protective Efficacy of the Conserved NP, PB1, and M1 Proteins as Immunogens in DNA- and Vaccinia Virus-Based Universal Influenza A Virus Vaccines in Mice. Clin Vaccine Immunol (2015) 22(6):618–30. doi: 10.1128/CVI.00091-15 PMC4446406 25834017

[38] Xu X Qian J Qin L Li J Xue C Ding J. Chimeric Newcastle Disease Virus-Like Particles Containing DC-Binding Peptide-Fused Haemagglutinin Protect Chickens From Virulent Newcastle Disease Virus and H9N2 Avian Influenza Virus Challenge. Virol Sin (2020) 35(4):455–67. doi: 10.1007/s12250-020-00199-1 PMC7462956 32274680

[39] Pillet S Racine T Nfon C Di Lenardo TZ Babiuk S Ward BJ. Plant-Derived H7 VLP Vaccine Elicits Protective Immune Response Against H7N9 Influenza Virus in Mice and Ferrets. Vaccine (2015) 33(46):6282–9. doi: 10.1016/j.vaccine.2015.09.065 26432915

[40] Ward BJ Makarkov A Séguin A Pillet S Trépanier S Dhaliwall J. Efficacy, Immunogenicity, and Safety of a Plant-Derived, Quadrivalent, Virus-Like Particle Influenza Vaccine in Adults (18-64 Years ) and Older Adults (≥65 Years ): Two Multicentre, Randomised Phase 3 Trials. Lancet (2020) 396(10261):1491–503. doi: 10.1016/S0140-6736(20)32014-6 33065035

[41] Gao X Wang W Li Y Zhang S Duan Y Xing L. Enhanced Influenza VLP Vaccines Comprising Matrix-2 Ectodomain and Nucleoprotein Epitopes Protects Mice From Lethal Challenge. Antiviral Res (2013) 98(1):4–11. doi: 10.1016/j.antiviral.2013.01.010 23416215

[42] Ramirez A Morris S Maucourant S D'Ascanio I Crescente V Lu IN. A Virus-Like Particle Vaccine Candidate for Influenza A Virus Based on Multiple Conserved Antigens Presented on Hepatitis B Tandem Core Particles. Vaccine (2018) 36(6):873–80. doi: 10.1016/j.vaccine.2017.12.053 29306508

[43] Sequeira DP Correia R Carrondo MJT Roldão A Teixeira AP Alves PM. Combining Stable Insect Cell Lines With Baculovirus-Mediated Expression for Multi-HA Influenza VLP Production. Vaccine (2018) 36(22):3112–23. doi: 10.1016/j.vaccine.2017.02.043 28291648

[44] Buffin S Peubez I Barrière F Nicolaï M-C Tapia T Dhir V. Influenza A and B Virus-Like Particles Produced in Mammalian Cells Are Highly Immunogenic and Induce Functional Antibodies. Vaccine (2019) 37(46):6857–67. doi: 10.1016/j.vaccine.2019.09.057 31590935

[45] Sari-Ak D Bahrami S Laska MJ Drncova P Fitzgerald DJ Schaffitzel C. High-Throughput Production of Influenza Virus-Like Particle (VLP) Array by Using VLP-Factory™, a MultiBac Baculoviral Genome Customized for Enveloped VLP Expression. High Throughput Protein Production Purif: Methods Protoc (2019) 2025:213–26. doi: 10.1007/978-1-4939-9624-7_10 31267455

[46] Kim K-H Lee Y-T Park S Jung Y-J Lee Y Ko E-J. Neuraminidase Expressing Virus-Like Particle Vaccine Provides Effective Cross Protection Against Influenza Virus. Virology (2019) 535:179–88. doi: 10.1016/j.virol.2019.07.008 PMC6946909 31310875

[47] Bouguyon E Goncalves E Shevtsov A Maisonnasse P Remyga S Goryushev O. A New Adjuvant Combined With Inactivated Influenza Enhances Specific CD8 T Cell Response in Mice and Decreases Symptoms in Swine Upon Challenge. Viral Immunol (2015) 28(9):524–31. doi: 10.1089/vim.2014.0149 26447972

[48] Hu J Liang Y Hu Z Wang X Gu M Li R. Recombinant Baculovirus Vaccine Expressing Hemagglutinin of H7N9 Avian Influenza Virus Confers Full Protection Against Lethal Highly Pathogenic H7N9 Virus Infection in Chickens. Arch Virol (2019) 164(3):807–17. doi: 10.1007/s00705-018-04142-4 30671655

[49] Smith T O'Kennedy MM Wandrag DBR Adeyemi M Abolnik C. Efficacy of a Plant-Produced Virus-Like Particle Vaccine in Chickens Challenged With Influenza A H6N2 Virus. Plant Biotechnol J (2020) 18(2):502–12. doi: 10.1111/pbi.13219 PMC6953208 31350931

[50] Wang Q Zhang Y Zou P Wang M Fu W She J. Self-Assembly M2e-Based Peptide Nanovaccine Confers Broad Protection Against Influenza Viruses. Front Microbiol (2020) 11:1961. doi: 10.3389/fmicb.2020.01961 32922379 PMC7457018

[51] Wang Y Deng L Gonzalez GX Luthra L Dong C Ma Y. Double-Layered M2e-NA Protein Nanoparticle Immunization Induces Broad Cross-Protection Against Different Influenza Viruses in Mice. Adv Healthc Mater (2020) 9(2):e1901176–e. doi: 10.1002/adhm.201901176 PMC6980908 31840437

[52] Ravin NV Blokhina EA Kuprianov VV Stepanova LA Shaldjan AA Kovaleva AA. Development of a Candidate Influenza Vaccine Based on Virus-Like Particles Displaying Influenza M2e Peptide Into the Immunodominant Loop Region of Hepatitis B Core Antigen: Insertion of Multiple Copies of M2e Increases Immunogenicity and Protective Efficiency. Vaccine (2015) 33(29):3392–7. doi: 10.1016/j.vaccine.2015.04.066 25937448

[53] Shokouhi H Farahmand B Ghaemi A Mazaheri V Fotouhi F. Vaccination With Three Tandem Repeats of M2 Extracellular Domain Fused to Leismania Major HSP70 Protects Mice Against Influenza A Virus Challenge. Virus Res (2018) 251:40–6. doi: 10.1016/j.virusres.2018.05.003 29730305

[54] Stepanova LA Mardanova ES Shuklina MA Blokhina EA Kotlyarov RY Potapchuk MV. Flagellin-Fused Protein Targeting M2e and HA2 Induces Potent Humoral and T-Cell Responses and Protects Mice Against Various Influenza Viruses a Subtypes. J Biomed Sci (2018) 25(1):33. doi: 10.1186/s12929-018-0433-5 29631629 PMC5891888

[55] Music N Reber AJ Kim M-C York IA Kang S-M. Supplementation of H1N1pdm09 Split Vaccine With Heterologous Tandem Repeat M2e5x Virus-Like Particles Confers Improved Cross-Protection in Ferrets. Vaccine (2016) 34(4):466–73. doi: 10.1016/j.vaccine.2015.12.023 PMC4713238 26709639

[56] Song B-M Kang H-M Lee E-K Jung SC Kim M-C Lee Y-N. Supplemented Vaccination With Tandem Repeat M2e Virus-Like Particles Enhances Protection Against Homologous and Heterologous HPAI H5 Viruses in Chickens. Vaccine (2016) 34(5):678–86. doi: 10.1016/j.vaccine.2015.11.074 PMC4721577 26691568

[57] Miller SM Cybulski V Whitacre M Bess LS Livesay MT Walsh L. Novel Lipidated Imidazoquinoline TLR7/8 Adjuvants Elicit Influenza-Specific Th1 Immune Responses and Protect Against Heterologous H3N2 Influenza Challenge in Mice. Front Immunol (2020) 11:406. doi: 10.3389/fimmu.2020.00406 32210973 PMC7075946

[58] Moriyama M Chino S Ichinohe T. Consecutive Inoculations of Influenza Virus Vaccine and Poly(I:C) Protects Mice Against Homologous and Heterologous Virus Challenge. Vaccine (2017) 35(7):1001–7. doi: 10.1016/j.vaccine.2017.01.025 28111142

[59] Wong PT Goff PH Sun RJ Ruge MJ Ermler ME Sebring A. Combined Intranasal Nanoemulsion and RIG-I Activating RNA Adjuvants Enhance Mucosal, Humoral, and Cellular Immunity to Influenza Virus. Mol Pharm (2021) 18(2):679–98. doi: 10.1021/acs.molpharmaceut.0c00315 32491861

[60] Jang SI Lillehoj HS Lee SH Lee KW Lillehoj EP Bertrand F. Montanide™ ISA 71 VG Adjuvant Enhances Antibody and Cell-Mediated Immune Responses to Profilin Subunit Antigen Vaccination and Promotes Protection Against Eimeria Acervulina and Eimeria Tenella. Exp Parasitol (2011) 127(1):178–83. doi: 10.1016/j.exppara.2010.07.021 20728439

[61] Zhu W-Z Wen Y-C Lin S-Y Chen T-C Chen H-W. Anti-Influenza Protective Efficacy of a H6 Virus-Like Particle in Chickens. Vaccines (2020) 8(3):465. doi: 10.3390/vaccines8030465 PMC7565593 32825685

[62] Chowdhury MYE Kim T-H Uddin MB Kim J-H Hewawaduge CY Ferdowshi Z. Mucosal Vaccination of Conserved Sm2, HA2 and Cholera Toxin Subunit A1 (CTA1) Fusion Protein With Poly Gamma-Glutamate/Chitosan Nanoparticles (PC NPs) Induces Protection Against Divergent Influenza Subtypes. Vet Microbiol (2017) 201:240–51. doi: 10.1016/j.vetmic.2017.01.020 28284616

[63] Wang SH Smith D Cao Z Chen J Acosta H Chichester JA. Recombinant H5 Hemagglutinin Adjuvanted With Nanoemulsion Protects Ferrets Against Pathogenic Avian Influenza Virus Challenge. Vaccine (2019) 37(12):1591–600. doi: 10.1016/j.vaccine.2019.02.002 30795941

[64] Deng L Chang TZ Wang Y Li S Wang S Matsuyama S. Heterosubtypic Influenza Protection Elicited by Double-Layered Polypeptide Nanoparticles in Mice. Proc Natl Acad Sci U S A (2018) 115(33):E7758–67. doi: 10.1073/pnas.1805713115 PMC6099848 30065113

[65] Deng L Mohan T Chang TZ Gonzalez GX Wang Y Kwon Y-M. Double-Layered Protein Nanoparticles Induce Broad Protection Against Divergent Influenza A Viruses. Nat Commun (2018) 9(1):359. doi: 10.1038/s41467-017-02725-4 29367723 PMC5783933

